# Canine Fecal Contamination in a Metropolitan Area (Milan, North-Western Italy): Prevalence of Intestinal Parasites and Evaluation of Health Risks

**DOI:** 10.1155/2014/132361

**Published:** 2014-11-17

**Authors:** Sergio Aurelio Zanzani, Anna Rita Di Cerbo, Alessia Libera Gazzonis, Marco Genchi, Laura Rinaldi, Vincenzo Musella, Giuseppe Cringoli, Maria Teresa Manfredi

**Affiliations:** ^1^Department of Veterinary Science and Public Health, Università degli Studi di Milano, 20143 Milan, Italy; ^2^Department of Veterinary Medicine and Animal Productions, Università degli Studi di Napoli “Federico II”, 80137 Naples, Italy

## Abstract

Intestinal parasites of dogs represent a serious threat to human health due to their zoonotic potential. Thus, metropolitan areas presenting high concentrations of pets and urban fecal contamination on public areas are at sanitary risk. Major aim of this survey was to determine prevalence of zoonotic parasites in dog fecal samples collected from public soil of Milan (north-western Italy). Differences in parasites prevalence distribution were explored by a geographical information system- (GIS-) based approach, and risk factors (human density, sizes of green parks, and dog areas) were considered. The metropolitan area was divided into 157 rectangular subareas and sampling was performed following a 1-kilometer straight transect. A total of 463 fecal samples were analyzed using centrifugation-flotation technique and ELISA to detect *Giardia* and *Cryptosporidium* coproantigens. A widespread fecal contamination of soil was highlighted, being fecal samples found in 86.8% of the subareas considered. The overall prevalence of intestinal parasites was 16.63%. Zoonotic parasites were found, such as *Trichuris vulpis* (3.67%), *Toxocara canis* (1.72%), *Strongyloides stercoralis* (0.86%), Ancylostomatidae (0.43%), and *Dipylidium caninum* (0.43%). *Giardia duodenalis* was the most prevalent zoonotic protozoa (11.06%), followed by *Cryptosporidium* (1.10%). Faeces from subareas characterized by broad green areas showed to be particularly prone to infection.

## 1. Introduction

Dogs are likely to transmit several zoonotic infections, among which those caused by intestinal helminths and protozoa can be of public concern [1, 2]. People living in urban areas, as cities or large metropolitan areas, are exposed to zoonotic parasites of canine source. Among the nematode species,* Toxocara canis* represents the major concern because it is well known to cause even severe infection in humans [3, 4]. Other zoonotic helminths, though often neglected, such as hookworms (*Ancylostoma caninum* and* Uncinaria stenocephala*) and* Trichuris vulpis*, are frequently recorded in dogs [5]. Moreover, dogs can harbor either host specific (C, D, F) or zoonotic assemblages (A and B) of* Giardia duodenalis*, the most frequent parasite affecting domestic carnivores in the last years [6–8]. Additionally, recent molecular-based surveys have shown that a few genotypes of* Cryptosporidium* spp. are responsible for most human cryptosporidiosis cases, including* C. canis* (dog type) [9, 10]. Nowadays, intestinal parasites of dogs represent an important concern for humans due to the increasing presence of these pets mainly in urban areas. The parasitic risks for humans are mostly posed by environmental fecal contamination. In fact, parasitic elements (eggs, larvae, cysts, and oocysts) excreted via canine fecal route can survive and be infective in the environment over a long time at different conditions [11]. Thus, not only can dog faeces deposited on public soil, parks, or gardens of cities represent an inconvenience, but it can be mostly a health risk as previously demonstrated [12–15].

Lombardy is the region of north-western Italy with the largest population of companion dogs, representing about 15% of their overall presence in Italy (data from National Companion Registry http://www.salute.gov.it/anagcaninapublic_new/AdapterHTTP). From 2003 to 2010, 834,075 dogs in Lombardy and approximately 100,000 of them in the city of Milan were recorded. High environmental fecal contamination still occurs in this area in spite of the fact that public parks now include off-leash fenced areas for a better control of the issue.

Major goal of this survey was to determine the prevalence of canine intestinal parasites in faeces spread on the ground of a large metropolitan area of north-western Italy (Milan) and deriving sanitary risks for humans. Further, differences in parasites prevalence among areas of the city were explored by a geographical information system (GIS) based approach and influences of some factors, such as human density and sizes of green and dog areas, were also considered.

## 2. Materials and Methods

### 2.1. Study Area and Sampling

The survey was carried out in Milan, a large metropolitan city located in the north-western Italian region of Lombardy (latitude: 45°40′N; longitude: 9°30′E). Milan covers an area of 183,77 km^2^ populated by 1,299,633 inhabitants (ISTAT 2010). The city presents a continental climate (temperature min–max: −4.0–15.6°C in the coldest month and min–max: 14.5–37.1°C in the warmest month) with an annual rainfall of 1,251 mm (average daily rainfall min–max: 0.50–6.45 mm) (ARPA, http://www.arpalombardia.it/arpa_splash/splash.asp). Milan has more than 21 million square meters of green urban areas organized in public parks presenting very different sizes (the biggest urban area reaches 6.4 million square meters) and is divided into 9 administrative districts (district area extension min–max: 967–3134 h). A GIS by the cadastral maps (1:1000) of the city of Milan (SIT, Cartographic Office of Milan Town Hall) was constructed, and a grid approach followed by transect sampling was used [13]. Then, the territory of Milan was divided into 157 equal, rectangular subareas of 1.6 km × 800 m; in each subarea, a 1-kilometer straight transect was identified along which a veterinary practitioner was instructed to collect 4 faecal samples. Each sampling point was georeferenced and maps with distribution of infected samples were created (ArcGIS 8.3). Further, the administrative district for each faecal sample was identified.

Out of 157 subareas only 138 could be investigated, 19 being inaccessible. The study was conducted between March and November 2010 and a total of 463 faecal samples were all collected in the early morning (before 9 am). All the samples collected derived from dog faeces (there are no foxes in urban Milan) and were fresh deposited (not more than a day).

### 2.2. Faecal Examination 

Macroscopic examination was firstly performed for the detection of proglottids of cestodes. Subsequently, each faecal sample was blended and divided into two aliquots. In order to detect parasite eggs and oocysts one aliquot was subjected to qualitative microscopic analysis by centrifugation-flotation technique with sucrose and sodium nitrate solution (specific gravity: 1360) [16]. The parasite eggs were differentiated according to their morphologic characteristics. The second aliquot was used to detect coproantigens of* G. duodenalis *and* Cryptosporidium* by enzyme linked immunosorbent assay (ELISA). For this purpose commercially available kits (RIDASCREEN* Giardia* and RIDASCREEN* Cryptosporidium*, R-Biopharm AG, Germany) were used following the manufacturer's recommended procedures. The negative and positive controls contained in the kits were used. Optical density (OD) of each sample was measured at 450 nm utilizing a microplate reader (Multiskan Ascent, Thermo Labsystems, Helsinki, Finland). OD values more than 10% above the calculated cutoff were considered positive. Sensitivity and specificity, respectively, were 100.0% and 99.6% for* Giardia *kit and 100.0% and 97.3% for* Cryptosporidium *kit.

### 2.3. Statistical Analysis

We defined prevalence according to Bush et al. [17]. Since prevalence of single taxa was too low for a risk factor analysis, data on infection with helminths and/or protozoa were also combined to the purpose. A sample was considered positive if tested positive for at least one species of parasites. Preliminary univariate logistic regression was performed considering the following independent variables: administrative district, human population density (inhabitant/km^2^), green area and dog area sizes (m^2^), percentage of green and dog areas calculated with respect to the administrative district size, and the number of dog areas for each administrative district. Data were inferred from ISTAT (2010) and Statistics Office of Milan Town Hall. Variables showing a* P* value <0.20 were included in the multivariate regression model. Backward elimination was used to determine which variables entered the final model, setting at 0.05 the level of significance to be included in the model. All statistical analysis was performed using SPSS v.19.0 (IBM Corp., Armonk, NY, USA).

## 3. Results

**Figure 1:**
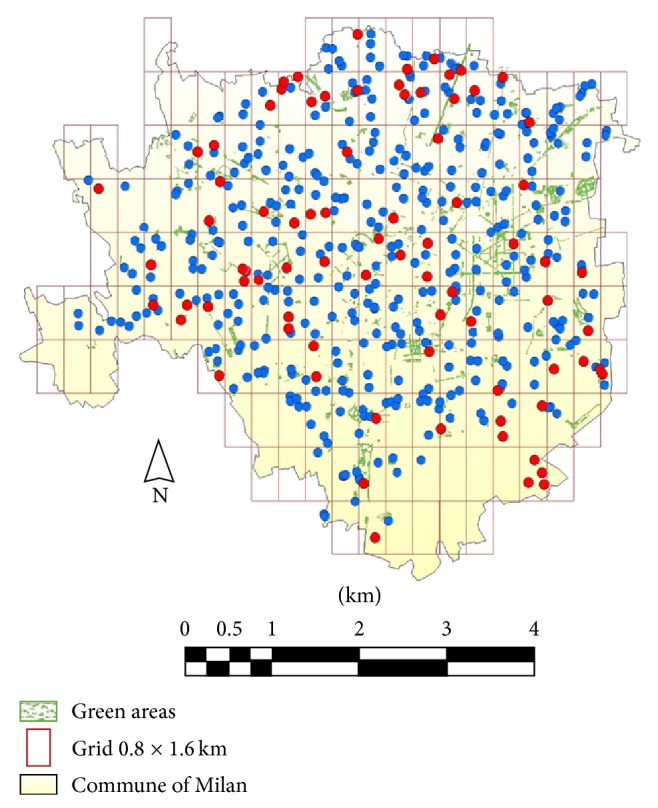
Distribution of dog faecal samples in the metropolitan area of Milan, north-western Italy. Location of negative (dot blue) and positive (dot red) dog faecal samples for parasitic elements.

Dog faecal samples (*n* = 463) were found and collected from 120 (86.8%) out of the 138 surveyed subareas. In most subareas (*n* = 110) 4 faecal samples were obtained from each (overall 440); in 5 subareas, 3 faecal samples (overall 15), and in 2 subareas, 2 faecal samples (overall 6). Finally, in 2 subareas, only 1 faecal sample in each (overall 2) was detected (Figure 1).

**Figure 2:**
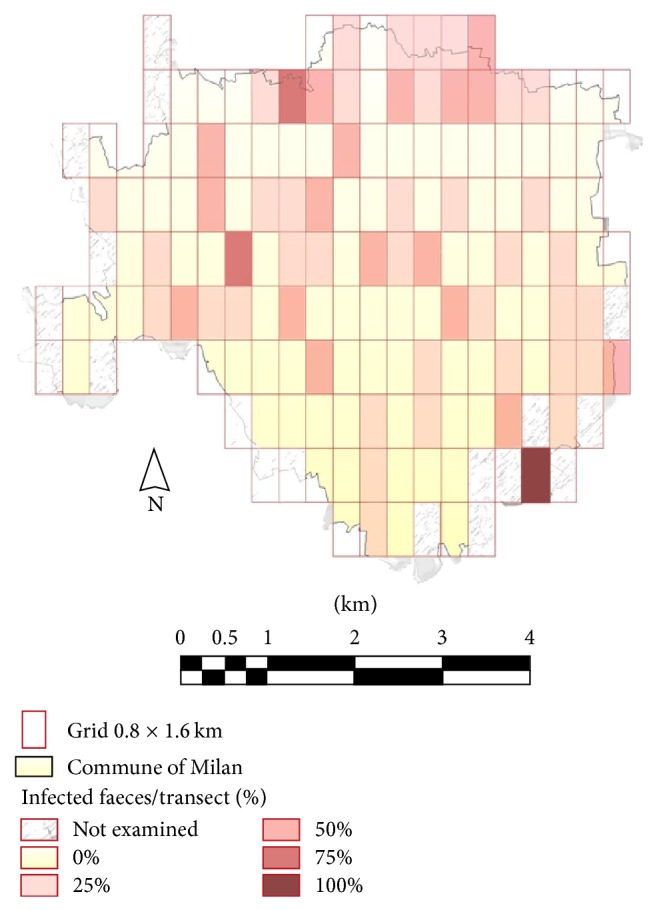
Proportion of dog faecal samples infected by parasites for each subarea of the metropolitan area of Milan (north-western Italy).

As shown in Table 1, both zoonotic and non zoonotic parasites were observed. Zoonotic parasites were the most frequent, though. As regards helminths, the following prevalences were found:* Trichuris vulpis* (3.7%),* Toxocara canis* (1.72%),* Strongyloides *spp.(0.86%), Ancylostomatidae (0.43%), and* Dipylidium caninum* (0.43%). In regard to protozoa, 11.06% of the samples showed coproantigens of* Giardia *and 11.10% were positive to* Cryptosporidium* coproantigens. Non zoonotic parasites, such as* Toxascaris leonina* and* Cystoisospora *with a prevalence of 0.64 and 0.21%, respectively, were also found. Mixed infections were detected in 11 faecal sample (2.4%, 95% CI = 1.3%, 4.2%); in all these samples* Giardia *was associated with* T. canis *(*n* = 2),* T. vulpis* (*n* = 7), and* T. leonina* (*n* = 2). Out of the 120 subareas considered, 57 (47.5%) were positive to parasitic elements.* G. duodenalis* was the most prevalent species detected (42 positive subareas).* T. vulpis *eggs were found in faecal samples from 16 subareas (13.3%).* T. canis *eggs were obtained in 6 subareas (5%); three of them were located in the center of Milan. In general, the spatial distribution of parasitic stages found in dog faeces did not show any correlation with particular areas of the city (Figure 2).

Results from the logistic regression analysis showed that the size of green areas present in each administrative district expressed by the proportion of green areas with respect to the administrative district area was the variable entered in the final multivariable model; then, the odds of a faecal sample being contaminated by parasitic elements increased by a multiplicative factor of 1.084 with a one percentage point increase of the green areas proportion in the administrative district (Table 2).

## 4. Discussion

The study demonstrated a widespread faecal contamination of Milan soil, being canine faecal samples found in 86.8% of the surveyed subareas. Further, as previously set during the study design, in most subareas (110 out of 138, i.e., 79.7%) four faecal samples could be collected. The intestinal parasites traced are consistent with the canine parasitic fauna and with the results obtained by previous surveys carried out in Italy [13, 18–21]. Most faecal samples contained elements of zoonotic parasites. Among nematodes, the most frequent zoonotic species were* Toxocara canis *and* Trichuris vulpis*; the former is worldwide known as triggering a mostly asymptomatic human infection or the* larval migrans* syndrome, a severe disease involving the SNC and/or the eye [4]. The latter can sustain a zoonosis of minor importance even though several cases have been described since 1956 when the first case in a child was reported by Hall and Sonnenberg [22]. In general,* T. vulpis* causesan unapparent disease, but symptomatic infections were also reported in humans [23–25]. Other parasites diffusing less relevant zoonoses were found, such as Ancylostomatidae and* Dipylidium caninum*.

Considering only helminth infections, prevalence (6.7%) of intestinal parasites in canine faecal samples collected from soil in Milan was similar to prevalence reported in a previous survey where eggs were found in 7% of dog faeces from public places, including parks, in Milan [18]. However, in both cases prevalence is lower than findings on pets sampled at Veterinary Clinics of Milan or in central Italy [20, 21]. As stated by Zanzani et al. [21], the difference in prevalence could be due to the kind of faecal samples collected from city soil that mainly included droppings voided by old dogs typically showing lower infection values than young ones. The currently reported prevalence rates of dog helminths are slightly different from those reported in a similar survey carried out in Naples (16.9%), a city in the south of Italy where stray dogs appear more widespread than in northern Italy (data from Italian Health Ministry, http://www.salute.gov.it/) [13]; nonetheless they are consistent with data from other investigated Italian urban areas even though differences in sampling must be considered [15, 26–28].

According to other authors* T. canis *eggs showed a low prevalence (1.72%) in faeces collected from soil [13, 14], whereas in the aforementioned survey carried out in Milan a prevalence of 5% and 5.5% was found in soil and faecal samples, respectively [18]. In contrast, 16.4% of soil samples collected in public parks of Madrid were demonstrated infected with* Toxocara* eggs by Dado et al. [14].

As regards protozoa,* G. duodenalis* was the most prevalent parasite according to other surveys [7, 14, 19, 29]. However, other data obtained by different analytical methods recorded lower prevalence values [18, 30–32]. Further, this protozoan seems largely spread among subareas of Milan unlike* Cryptosporidium *whose coproantigens were found in a very low number of samples and subareas. The prevalence value of* Cryptosporidium *is lower than those recently found in Spain varying from 9% throughout 17.6% [14]. Regarding sanitary risks posed by these protozoa, in dogs from Lombardy* G. duodenalis* assemblages C and D (i.e.,* Giardia canis*) were previously isolated, but zoonotic assemblages can be hosted by dogs [8, 21]. Moreover, both* C. canis *and* C. parvum* were identified in the faeces of two dogs from Milan and underwent clinical examination (Manfredi et al., unpublished data). Accordingly, veterinarians should pay more attention to these potentially zoonotic protozoa and improve both their diagnostic and control levels using appropriate methods and due treatments.

Finally, the main risk factor associated with the presence of parasitic stages in dog faecal samples resulted to be the extension occupied by green areas within an administrative district area. In fact, faecal samples collected from administrative districts characterized by a large proportion of green areas were more positive than faeces from administrative districts whose territory showed a smaller proportion of green areas. Therefore, it can be inferred that green areas may contribute to maintain environmental contamination of public areas by canine faeces and deriving health risks posed by dog parasites. In order to prevent sanitary risks for humans, off-leash fenced areas for dogs to be set in public parks, small gardens, or traffic islands have been claimed in each district. However, the presence of fenced areas, as only control measure of dog parasites, is not able to eradicate the problem since any reserved areas can reasonably become a reservoir of parasites for dogs soiling it as no appropriate treatments are available to free them from parasites. Nowadays a regular control of dog parasites coupled with an appropriate laboratory diagnosis is needed in order to prevent the diffusion of zoonotic parasites in public areas. Owner should be educated to collect dropping voided by their own pets on public areas and to check the parasitic status of their dogs regularly. Other important risk factors associated with endoparasites in dogs from different urban areas resulted to be both animal age and their sharing the same house with other dogs. Thus, there is a strong need for parasitic monitoring of dogs younger than 12 months and of those living with other pets [21, 33–35]. Particularly, younger dogs are more exposed to* Toxocara canis* infections that they can acquire by several routes such as transplacental and transmammary routes by migrating larvae, ingestion of embryonated eggs from the environment or finally by somatic larvae via paratenic hosts. It should be further considered that even though dogs older than 12 months show a parasitic spectrum slightly different from that of younger animals, they themselves may be infected by zoonotic parasites [21]. Last, but not least, owners should be helped to properly consider canine zoonotic parasites. As demonstrated by a survey carried out in Lombardy, a large part of them (50.8%) are not aware about the fact that gastrointestinal (GI) parasites of their dogs do represent a risk to human health [21, 36]. Veterinarians should be more determined in playing their key role in this educational step and in submitting dogs to periodic coprological examination as stated by the international guidelines for control of canine parasites (ESCAAP) as well as more careful in improving the diagnosis of GI parasites in the consideration that they infect nearly 45% of dogs presenting GI signs which urges a differential diagnosis [21, 36].

## 5. Conclusions 

According to the results of this survey, canine faecal samples from public areas in Milan show a relatively high presence of intestinal parasites, among which zoonotic parasites were found most frequently (*T. canis*,* T. vulpis*, Ancylostomatidae, and* G. duodenalis*). In spite of control measures against environmental faecal pollution recently set by local authorities, such as off-leash fenced dog areas within public green areas, the issue is still on. In fact, it requires further and continuous monitoring and control of gastrointestinal parasites in owned dogs to which veterinarians can contribute by properly informing and educating owners about a correct behavior in defense of the health of their pets and of other companion animals they can come in contact with in the urban scenarios.

## Figures and Tables

**Table 1:** Prevalence (%) and 95% confidence interval (CI) of intestinal parasites in 463 dog faecal samples and 120 subareas in Milan (north-western Italy).

	Faecal samples		Subareas	
	*n*	% (95 CI)	*n*	% (95 CI)
*Toxocara canis *	8	1.72 (0.88–3.37)	6	5.00 (2.05–11.02)
*Toxascaris leonina *	3	0.64 (0.22–1.89)	3	2.50 (0.65–7.68)
*Ancylostomatidae *		0.43 (0.12–1.56)	2	1.67 (0.29–6.5)
*Trichuris vulpis *	17	3.67 (2.22–5.93)	16	13.33 (8.05–21.04)
*Strongyloides *spp.	4	0.86 (0.28–2.35)	4	3.33 (1.07–8.82)
*Dipylidium caninum *	2	0.43 (0.12–1.56)	2	1.67 (0.29–6.5)
*Cystoisospora *sp.	1	0.21 (0.04–1.22)	1	0.83 (0.04–5.23)
*Giardia duodenalis *	50	11.06 (8.49–14.29)	42	35.00 (26.67–44.30)
*Cryptosporidium *sp.	5	1.10 (0.47–2.55)	3	2.50 (0.65–7.68)
Overall prevalence	77	16.63 (13.52–20.29)	57	47.50 (38.38–56.78)

**Table 2:** Final multivariate analysis of risk factors associated with intestinal parasites in dog faecal samples collected in public areas of Milan (north-western Italy).

Variable	Risk factor	Cases	Odd ratio	95% CI^*^	*P* value
Green areas	Proportion of territory of the administrative district occupied by green areas	463	1.084	1.030–1.140	0.002

^*^Confidence interval.
